# Factors Affecting the Life Satisfaction of Older People with Care Needs Who Live at Home

**DOI:** 10.3390/geriatrics7050117

**Published:** 2022-10-18

**Authors:** Yuka Misu, Shintaro Hayashi, Nobuhiko Iwai, Taisei Yamamoto

**Affiliations:** 1 Graduate School of Rehabilitation, Kobe Gakuin University, Kobe 651-2180, Japan; 2 Department of Physical Therapy, Faculty of Rehabilitation, Morinomiya University of Medical Sciences, Osaka 559-8611, Japan; 3 Department of Physical Therapy, Faculty of Rehabilitation, Kobe Gakuin University, Kobe 651-2180, Japan; 4 Department of Physical Therapy, School of Health Sciences, Tokyo International University, Kawagoe 350-1197, Japan

**Keywords:** life satisfaction, older people with care need, participation, occupational gaps, sense of coherence, environmental factors

## Abstract

The number of older people is increasing rapidly, and the number of older people with care needs who live at home is also increasing in Japan. Maintaining their life satisfaction has been a primary challenge. This study aimed to identify factors affecting the life satisfaction of older people with care needs. The study was conducted among older people using homecare services; 126 participants (mean age, 79.33 ± 7.51 years, 54 male) were included in the analysis. Logistic regression analysis with adjustment for age, sex, and economic status was conducted with life satisfaction as the objective variable and the Japanese version of occupational gaps questionnaire (OGQ-J), sense of coherence, functional independence measure, and environmental factors as explanatory variables. The variables that significantly affected life satisfaction were the OGQ-J (*p* = 0.0352, OR 0.90, 95% CI 0.81–0.99) and environmental factors (*p* = 0.0083, OR 4.41, 95% CI 1.52–14.11). This study’s results indicate the importance of focusing on environmental factors and facilitating the participation of older people with care needs in activities they want to do to maintain and improve their life satisfaction.

## 1. Introduction

In September 2021, approximately 36.2 million people in Japan were >65 years old. The ratio of older people to the total population reached 28.9%, a record high [1]. The aging rate in Japan is the highest worldwide, and ensuring the maintenance of a better life for them is challenging. Achieving a better life for older people is an ongoing challenge because the populations in some countries are expected to age at a faster rate than that in Japan.

In 2016, the Japanese government surveyed the attitudes of Japanese people aged ≥40 years toward an aging society [2]. The results showed that 73.5% of the respondents wished to receive care in a familiar home with family members or nursing care services when necessary. The Japanese government is promoting medical and nursing care at home to enable older people to live in their homes even when they require the highest level of nursing care [3]. The number of older people with care needs living at home is expected to keep increasing [4], and they will need more support and care to continue living a satisfactory life in their homes until death.

In 2020, the Japanese government conducted the survey “International Comparative Survey on the Lives and Attitudes of the Elderly”, which reported that approximately 50% of the older people in the United States, Germany, and Sweden, whereas only 20% of the older people in Japan reported being “satisfied with their lives” in terms of the current life situation [5]. This report indicates that older people in Japan are less satisfied with their lives than those in other countries. In addition, previous studies of older Japanese people have reported that those with declining physical functions have lower life satisfaction [6], and those with lower levels of independence in activities of daily living (ADL) have lower life satisfaction than those with high levels of independence [7]. There are challenges in maintaining and improving the life satisfaction of older Japanese people with care needs. Previous studies have reported that life satisfaction among older people is related to their abilities and function, such as physical health [8], level of care required, bedridden state [9], and degree of ADL independence [7,9].

However, previous studies on survivors of stroke and individuals with spinal cord injuries reported that the level of disability is not related to life satisfaction [10,11]. Quality of life (QOL) varies widely even in the same level of disability, and physical function and ADL ability are not sufficient to explain QOL [12]. In recent years, many reports have focused on participation as a factor related to life satisfaction. The World Health Organization (WHO) defines “participation” as involvement in situations of life [13]. Participation is considered to have a positive influence on health and well-being and is vital for all humans [14]. Hartman-Maeir et al. [15] reported that life satisfaction was more strongly associated with participation status than with the level of ADL independence 1 year after stroke onset. Bergstrom et al. [16] reported that, in a study of people 5 years after stroke onset, those who did not have participation constraints obtained significantly higher levels of life satisfaction than those with participation constraints. This previous study focused on participation in activities that the person wants or needs to perform, and it did not focus on the performance status or ability of the participants in performing these activities. The gap between what the participant wants to do and what they actually do was defined as the occupational gap [17], and previous studies of stroke survivors have reported that individuals who have fewer occupational gaps show a tendency of achieving a higher degree of satisfaction in life [18,19].

The International Classification of Functioning, Disability, and Health (ICF) is a framework reflecting the dynamic, nonlinear interaction between health conditions, activity and participation, body functions, and structures as well as personal and environmental factors [13]. According to the ICF, the interaction of environmental and individual factors may promote activity and participation without improving body functions and structures [13]. Therefore, environmental factors are important for the participation and life satisfaction of older people with reduced physical and mental functions [20,21].

Personal factors influencing life satisfaction have focused on the sense of coherence (SOC), a core concept in salutogenesis [22,23]. In salutogenesis, aging is inevitable, but humans can live a vital life until death, and health can be generated even in the states of disease and decrease in function [23]. Based on salutogenesis, SOC is an important personal factor in maintaining life satisfaction among older people with care needs.

These findings are indicative of the fact that to maintain and improve the life satisfaction of older people with care needs, whose physical functions are difficult to improve or declining gradually, it is important to administer rehabilitation that focuses on not only physical functions but also environmental factors and personal factors as well as activities and participation. Furthermore, assessing not only the ability to participate and the implementation status of the activities but also the participation in the activities they want to perform and to understand how it effects their life satisfaction is of paramount importance. The occupational gaps questionnaire (OGQ) was developed to measure the occupational gap [17,18]. However, the Japanese version of the OGQ (OGQ-J) has not yet been developed, and information on the occupational gap of older people with care needs is lacking. It is imperative to clarify these issues in order to suggest appropriate rehabilitation methods to improve the life satisfaction of elderly people with care needs.

Therefore, the purpose of this study was to clarify how environmental factors, SOC, functional independence, and participation influence the life satisfaction of older people with care needs who live at home and to validate psychometrically the OGQ-J, which measures occupational gaps.

## 2. Materials and Methods

### 2.1. Participants

**Figure 1:**
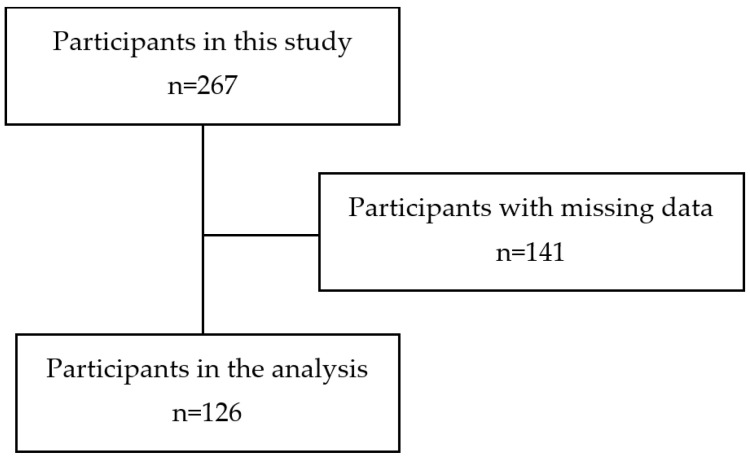
Flow chart of participant selection.

In this cross-sectional study, the participants were recruited from older people who were certified as needing support and nursing care under long-term care insurance and were using delivered homecare services and daycare services at home. Participants were individuals aged >65, living at home for at least 3 months, with preserved cognitive function, and could answer a self-rating questionnaire. Whether the participant had the cognitive function to respond to the self-rating questionnaire was determined by a physical therapist or occupational therapist who was fully aware of the participant’s cognitive status through daily care. Questionnaires were distributed to 267 individuals who consented to participation in this study. Of these individuals, 126 (47.2%) filled out all items in the questionnaires and were included in the analysis (Figure 1).

The study complied with the guidelines of the Declaration of Helsinki and was approved by the Ethics Committee of Kobe Gakuin University (IRB: 20–34). All participants provided informed consent.

### 2.2. Instruments

The questionnaire collected details such as the participant’s age, sex, family structure (living alone, living with a partner, and living with others), educational background (graduated from elementary school, junior high school, high school, and college or university), and perceived economic status (wealthy, normal, and poor).

The Life Satisfaction Checklist (LiSat-11) [24] was used to assess life satisfaction. The LiSat-11 consists of 11 items that can assess overall and domain-specific life satisfaction. The first question on the LiSat-11, “Life as a whole is …?”, is a relatively general question to comprehensively assess life satisfaction, and the validity of using only this question has been confirmed [25]. In the present study, the overall life satisfaction was used to assess participant’s life satisfaction, following the same procedure as mentioned in a previous study [16]. The scale is a six-point self-rating scale, ranging from 1, “very dissatisfied”, to 6, “very satisfied”; a score of 1–3 indicated “unsatisfied”, and a score of 4–6 indicated “satisfied”. The psychometrically validated LiSat-11 Japanese version was used in this study [26].

The OGQ-J was used to assess participation in activities. The original version of OGQ consists of 30 activities, which were instrumental ADL (IADL), leisure activities, social activities, and work or work-related activities. Participants were asked “Do you perform this activity?” and “Do you want to perform this activity?” for each item, with “yes” or “no” as responses for each question [18]. The OGQ has been psychometrically validated and is considered a functional instrument for measuring occupational gaps [18,27].

For this study, the OGQ was translated into Japanese using standard linguistic validation procedures [28] and adapted to Japanese culture [29]. Three occupational therapists, one psychiatrist, one physiotherapist, and the original author helped create the OGQ-J. The OGQ-J consists of 30 items, with the addition of “dressing up” as one of the “social activities” items, which was not included in the original version. In this study, the total number of activities that answered “no” to the question “Do you perform this activity?” and “yes” to the question “Do you want to perform this activity?” was used to indicate the occupational gap.

The 13-item SOC scale (SOC-13) was used to assess SOC [22]. The SOC-13 is a self-administered questionnaire developed by Antonovsky and has been tested for reliability and validity [22,30]. The SOC-13 consists of 13 questions about how one feels about life, and questionnaire items are rated on a 7-point Likert scale. Higher SOC scores indicate a high capacity for adaptation in their life. The Japanese version of the SOC-13 items, which has been confirmed to be reliable and valid, was used [31].

This study comprehensively examined environmental factors considered important to the life functions of older people with care needs. The environmental domain of the WHOQOL-BREF [32], Comprehensive Environmental Questionnaire [33], and Craig Hospital Inventory of Environmental Factors [34] were used as references in selecting assessment items. The WHO refers to environmental factors make up the physical, social, and attitudinal environment in which people live and conduct their lives [13]. In this study, the questionnaire on environmental factors consisted of 12 items, which included four items on the physical environment (mobility at home, comfort level at home, ease of going out, and transportation around the house), three items on the attitudinal environment (relationship with family, friends and acquaintances, and local residents), and five items on the social environment (whether the person receives the necessary care and support, whether care services are accessible, satisfaction with the services they use, whether the environment is conducive for participation in leisure activities, and whether the environment provides access to necessary stuff and information). The questionnaire on environmental factors is rated on a 5-point scale, ranging from “1” indicating applicable to “5” meaning very applicable; higher scores indicate a finer environment in the domain. For three items of the attitudinal environment, the mean scores were calculated excluding the items related to relationships with family and friends; this is because the person who has no family member or friend cannot answer these questions.

The functional independence measure (FIM) [35] was used to assess the degree of functional independence, which consists of 13 motor items and 5 cognitive items and is rated on a 7-point scale, in which 1 indicates total assistance, and 7 means independence. A higher score on the FIM indicates greater functional ability. The FIM reportedly has high reliability and validity and is widely used in rehabilitation [36]. The total score for each motor and cognitive item was calculated.

### 2.3. Data Collection

Participants were recruited at the collaborating facilities and selected by a physical therapist or occupational therapist working at these facilities. Participants who consented to the study were given a handout with a self-rating questionnaire in which demographic data, LiSat-11, OGQ-J, environmental factors, and SOC were surveyed.

Participants anonymously responded to survey form, and the completed forms were sent to the authors via mail. The FIM used to assess functional independence was assessed by an occupational therapist, physical therapist, or nurse who regularly observed the participants’ activities of daily living and was collected and returned by mail separately from the participants’ survey forms.

### 2.4. Statistical Analysis

#### 2.4.1. Psychometric Validation of the OGQ-J

Before examining factors affecting life satisfaction, the Rasch model was used to validate the internal scale validity of the OGQ-J. The Rasch model is increasingly used in rehabilitation medicine for developing and evaluating the psychometric properties of new and existing assessments [37]. They are also used to examine whether items from tests or questionnaires measure unidimensional constructs. Using probabilistic transformation techniques, Rasch computer programs are used to convert the raw item scores from a test or questionnaire into equal-interval measures commonly referred to as logits [37]. In this study, the acceptance criteria for item fitness were determined to support the internal scale validity and person-response validity with item and person infit MnSq values of <1.4 logit and *z* values of <2.0, as per previous studies [18,19,27]. In this study, item and person fit indices and the unidimensionality of the OGQ-J were analyzed by principal component analysis of residuals. The analyses were conducted in the Rasch computer program WINSTEPS version 5.24.

#### 2.4.2. Factors Affecting Life Satisfaction

Participants were assigned into two groups: an unsatisfied group with a life satisfaction of ≤3 and a satisfied group with a life satisfaction ≥ 4. Each variable identified from the survey was compared between the two groups, and the variables were examined for entry into a logistic regression analysis. Age, SOC-13, and environmental factors were analyzed using the *t*-test, sex and family structure were analyzed by the χ² test, and other items were analyzed using the Mann–Whitney test. Multicollinearity for the logistic modeling was checked using Spearman’s rank correlation coefficients between variables. Logistic regression analysis (forced entry method) was conducted with life satisfaction as the objective variable, variables determined by univariate analysis as explanatory variables, and age, sex, and economic status as adjustment variables. Statistical analysis was conducted using R version 4.1.1 with a significance level of 5%.

## 3. Results

### 3.1. Participants

Table 1 shows the characteristics of the study participants. The mean ± standard deviation (SD) of age was 79.33 ± 7.51 years; 54 (42.9%) were male, and 72 (57.1%) were female. Regarding family structure, 32 (25.4%) were living alone, 44 (34.9%) were living with a partner, and 50 (39.7%) were living with others. Overall, 3 respondents (2.4%) graduated from elementary school; 41 (32.5%) from junior high school; 56 (44.4%) from high school; and 26 (20.6%) from college or university. For economic status, 11 respondents (8.7%) considered themselves “wealthy”, 108 (85.7%) as “normal”, and 7 (5.6%) as “poor”. In comparing two groups, including 141 respondents with missing data and 126 participants included in the analysis, no significant differences were found in any of the items in terms of basic attributes in the returned survey forms.

In the analysis of LiSat-11, 2 (1.6%) were very dissatisfied, 10 (7.9%) dissatisfied, 26 (20.6%) rather dissatisfied, 51 (40.5%) rather satisfied, 33 (26.2) satisfied, and 4 (3.2) very satisfied of their current life. The median of occupational gaps as measured by the OGQ-J was 4 (range, 0–22). The SOC-13 core was 60.23 ± 11.65. The FIM motor score was 82.61 ± 9.35. The FIM cognitive score was 33.11 ± 3.27, and the score on the environmental factor questionnaire was 3.67 ± 0.52.

### 3.2. Results of Psychometric Validation of the OGQ-J

The results of the survey of participants’ occupational gaps using the OGQ-J revealed that in terms of the activity that they do not do but they wanted to do, most of the participants selected “traveling for pleasure” (*n* = 48, 38.1%), followed by participating in cultural activities (*n* = 38, 30.2%), “helping and supporting others” (*n* = 34, 27.0%), “participating in outdoor activities” (*n* = 34, 27.0%), and “participating/taking interest in sports” (*n* = 33, 26.2%).

The results of Rasch analysis showed that the distribution of the occupational gap by items ranged from −1.27 to 1.32 logits (mean = 0.00, SD = 0.66). The distribution of the occupational gap by participants ranged from −4.77 to 1.30 logits (mean = −1.97, SD = 1.44), and 114 (90.4%) participants fit the Rasch model. Cronbach’s alpha coefficient was 0.89. The indices of person reliability, person separation, item reliability, and item separation were 0.68, 1.45, 0.82, and 2.17, respectively. A principal component analysis of residuals revealed that the OGQ scale could explain 38.9% of the variance in the data set.

### 3.3. Factors Affecting Life Satisfaction

Regarding life satisfaction, 38 (30.2%) were allocated to the unsatisfied group and 88 (69.8%) to the satisfied group according to their LiSat-11 scores. Table 2 shows a comparison of characteristics between the unsatisfied and satisfied groups. The unsatisfied and satisfied groups were compared for each variable, and significant differences were found in the following items: economic states (*p* = 0.0079), OGQ-J (*p* < 0.001), SOC-13 (*p* = 0.0032), environmental factors (*p* < 0.001), FIM motor items (*p* = 0.0167), and FIM cognitive items (*p =* 0.0383). No strong correlations that affect multicollinearity were found for each of the variables in the items that were significantly different in the univariate analysis. Logistic regression analysis that was adjusted for age, sex, and perceived economic status using the forced entry method was conducted with life satisfaction as the objective variable and OGQ-J, SOC-13, FIM motor and cognitive items, and environmental factors as explanatory variables. Significant differences were found for OGQ-J (*p* = 0.0352, odds ratio (OR) 0.90, 95% confidence interval (CI) 0.81–0.99) and environmental factors (*p* = 0.0083, OR 4.41, 95% CI 1.52–14.11). The discriminant accuracy rate was 80.16% (Table 3).

## 4. Discussion

### 4.1. Validity of the OGQ-J

The OGQ-J was developed and validated to use the OGQ, which measures the occupational gap, as an assessment of participation in this study. The Rasch analysis revealed that all items of the OGQ-J showed goodness of fit to the Rasch model (MnSq values of <1.4 logit and *z* values of <2.0), and it confirmed the validity of the internal scale. In previous studies that have validated the OGQ, all items have shown goodness of fit for the Rasch model. The 30 items of the OGQ-J are useful for measuring the occupational gap in older people with care needs. Human response validity is not considered significantly compromised if the misfit is <5% [17,19,38]. The results of this study were slightly below the criteria, with 12 of 126 (9.5%) not fitting. The 12 participants who did not fit the Rasch model did not report any occupational gap and did not show the same responses for each activity item. Although these 12 participants were not fitted for the Rasch model, the OGQ-J was able to measure the characteristics of the occupational gap in older people with care needs. The Rasch model states that the person and item domains must detect at least two distinct groups each, and a person and item separation index 1.5 or higher is desirable [39]. This study showed person and item separation indices of 1.45 and 2.17, respectively, approximating previous studies’ values (1.67–1.82 and 2.88–3.32) [18,19,27]. The OGQ-J was able to separate persons and items into two distinct groups, indicating that it can find a wide range of people with an occupational gap. Unidimensionality is also further supported if the measurements obtained from the OGQ-J responses explained 50% of the total variance of the data, with <5% of the variation not explained by the first contrast [40]. In this study, principal component analysis of standardized residuals of the non-homogeneous sample showed that 38.9% of the total variance was explained by the Rasch dimension. This was lower than the criterion we set. The unexplained variance of the first contrast explained 6.5% of the residuals. This result does not fully satisfy the criteria for unidimensionality but follows similar trends to those reported by previous studies [19,27]. The OGQ-J may have subdimensions depending on the area of activity, which needs to be interpreted by the domain.

### 4.2. Factors Affecting Life Satisfaction

This study was conducted to determine how functional independence, participation, environmental factors, and SOC affect the life satisfaction of older people with care needs. The results revealed that comprehensive environmental factors and participation in activities that the person wants to do have greater effects than functional independence and SOC have on life satisfaction among older people with care needs.

In 2002, the WHO developed the concept of “active aging” as a response to the progress of global aging [41]. Active aging places considerable value on individuals’ participation in activities that they find meaningful [42]. The study focused on participation in activities they wanted to do rather than on their ability to participate or their execution of those activities. The results of this study show that participation in activities they desired affects their life satisfaction; additionally, most of the activities that participants “do not do but want to do” are social and leisure activities in OGQ-J. The results of this study confirm the importance of the concept proposed by WHO and suggest that even older people with care needs may maintain a better level of life satisfaction by promoting participation in activities that “they want to do”. Even though a person may have care needs, participation in social and leisure activities is an important perspective that can lead to further improvement in life satisfaction.

The results of this study suggest that comprehensive environmental factors may be a significant factor that influences life satisfaction. The results support previous studies reporting that physical/structural barriers of environmental factors are strongly related to satisfaction with participation among older people using homecare services [43]. The life functions of older people with reduced mental and physical function are considered more influenced by environmental factors. In this study, the participants were older people with care needs and had some decline in their physical and mental functions. Therefore, environmental factors may have a stronger influence on life satisfaction. In this study, three environmental aspects were comprehensively assessed based on the ICF definition: physical environment, attitudinal environment, and social environment [13]. Environmental factors that were assessed comprehensively demonstrated significant effects on the life satisfaction of older people with care needs. The study indicated the importance of comprehensive assessment and support for the three aspects of environmental factors, namely physical environment (such as the environment in and around the home), human environment (such as building good relationships with family and friends), and social environment (such as the accessibility of health and social care services), to improve the life satisfaction of older people with care needs.

Finally, SOC score was significantly higher in the satisfied group than in the unsatisfied group in univariate analysis, but no significant effect was found in multivariate analysis. In previous studies, people with higher SOC scores reported having a higher QOL [30,44] and life satisfaction [45,46], which was different from the predicted results in the present study. SOC increases with age and remains stable in the presence of chronic illness and disability [44]. Therefore, it was considered insufficient to explain life satisfaction in this study. In addition, this study examined factors that affect life satisfaction in a statistical model, including environmental factors and participation, which may have reduced the influence of SOC. The SOC score for an individual is a more or less constant value during adult life, being established by the end of the second decade of life, and with only minor and temporary changes in response to major changes in patterns of life experiences [47]. The SOC of older people is considered stable and not easily changed. Thus, it is difficult to expect change through support and intervention. The results of this study suggest that even those with low SOC scores can be expected to have improved their life satisfaction by improving environmental factors and promoting participation in desired activities.

In the rehabilitation of older adults with care needs, finding out what activities the person wants and needs to perform and considering methods to achieve them will lead to increase their life satisfaction rather than directly approaching their physical functions. The only facilitating factor for activity and participation is environmental factors in the ICF [13]. Participation may be promoted by modifying the environment even if physical function has declined and is not expected to improve.

### 4.3. Limitations of the Study

In this cross-sectional study, the results can be interpreted as associations between each factor at the time but not their causal relationships. A more precise analysis of the effects of environmental factors and participation on life satisfaction would be possible by analyzing longitudinal data, such as panel data analysis, to show causal relationships between each factor.

The study participants included those with preserved cognitive function who could complete the self-administered survey form. Participants with missing data in the survey form were excluded from the analysis. Therefore, the participants were considered to have a higher level of functional independence than we expected. Generalizing the results of this study requires designing the survey items and methods and validating the inclusion in the analysis of people with lower levels of functional independence than those in this study. However, to our knowledge, this study is the first to examine the influence of functional independence, participation in the activities they want to do, environmental factors, and SOC on the life satisfaction of older people with care needs in Japan. The study revealed key findings for maintaining and improving the life satisfaction of older people who have difficulty improving their physical and mental functions.

## 5. Conclusions

This study examined the effects of functional independence, participation, environmental factors, and SOC on life satisfaction among older people with care needs living at home. The life satisfaction of older people with care needs was explained by comprehensive environmental factors that consist of the physical, the attitudinal, and the social and participation in the activities they want to do. The effect of functional independence and SOC on life satisfaction was not significant. This study showed that it is important to provide comprehensive support for the physical, human, and social environment to improve the life satisfaction of older people with care needs. The results of this study suggest that to maintain and improve their life satisfaction among older people with care needs, it is important to focus on environmental factors and support them to promote participation in desired activities rather than improve their functional independence.

## Figures and Tables

**Table 1:** Characteristics of the study participants (*n* = 126).

Variables	*n*, Mean, Median	%, SD, Range
Age, Mean (SD), years	79.33	(7.51)
Sex, *n* (%)		
Male	54	(42.9)
Female	72	(57.1)
Family structure, *n* (%)		
Living alone	32	(25.4)
Living with a partner	44	(34.9)
Living with others	50	(39.7)
Education background, *n* (%)		
Elementary school	3	(2.4)
Junior high school	41	(32.5)
High school	56	(44.4)
College or university	26	(20.6)
Economic status, *n* (%)		
Wealthy	11	(8.7)
Normal	108	(85.7)
Poor	7	(5.6)
LiSat-11 ^1^, *n* (%)		
Very dissatisfied	2	(1.6)
Dissatisfied	10	(7.9)
Rather dissatisfied	26	(20.6)
Rather satisfied	51	(40.5)
Satisfied	33	(26.2)
Very satisfied	4	(3.2)
OGQ-J ^2^, Median (Range)	4	(0–22)
SOC-13 ^3^, Mean (SD)	60.23	(11.65)
FIM ^4^, Mean (SD)		
Motor items	82.61	(9.35)
Cognitive items	33.11	(3.27)
Environmental factors, Mean (SD)	3.67	(0.52)

^1^ LiSat-11, Life Satisfaction Checklist; ^2^ OGQ-J, Japanese version of Occupational Gaps Questionnaire; ^3^ SOC-13, 13-item Sense of Coherence; ^4^ FIM, Functional Independence Measure.

**Table 2:** Comparison of characteristics between unsatisfied group and satisfied group.

	Unsatisfied Group (*n* = 38)		Satisfied Group (*n* = 88)		*p*-Value
Age, Mean (SD), years	77.55	(7.82)	80.10	(7.82)	0.062
Sex, *n* (%)					0.614
Male	15	(39.5)	39	(44.3)	
Female	23	(60.5)	49	(55.7)	
Family structure, *n* (%)					0.291
Living alone	9	(23.7)	23	(26.1)	
Living with a partner	17	(44.7)	27	(30.7)	
Living with others	12	(31.6)	38	(43.2)	
Education background, *n* (%)					0.775
Elementary school	0	(0)	3	(2.4)	
Junior high school	14	(36.8)	27	(21.4)	
High school	17	(44.7)	39	(31.0)	
College or university	7	(18.4)	19	(15.1)	
Perceived economic status, *n* (%) **					0.079
Wealthy	1	(2.6)	10	(7.9)	
Normal	32	(84.2)	76	(60.3)	
Poor	5	(13.2)	2	(1.6)	
OGQ-J ^1^, Median (Range) **	7	(0–22)	2	(0–17)	<0.001
SOC-13 ^2^, Mean (SD) **	55.63	(9.71)	62.21	(11.9)	0.0032
FIM ^3^, Mean (SD)					
Motor items *	79.47	(10.86)	83.98	(8.33)	0.0167
Cognitive items *	31.92	(4.77)	33.62	(2.20)	0.0383
Environmental factors, Mean (SD) **	3.36	(0.47)	3.81	(0.48)	<0.001

^1^ OGQ-J, Japanese version of the Occupational Gaps Questionnaire; ^2^ SOC-13, 13-item Sense of Coherence; ^3^ FIM, Functional Independence Measure; * *p* < 0.05, ** *p* < 0.001

**Table 3:** Binary logistic regression analyses of variables potentially associated with life satisfaction (*n* =126).

	Estimate	*p*-Value	Odds Ratio	95% CI	
				Lower	Upper
OGQ-J ^1^ *	−0.107	0.0352	0.90	0.81	0.99
SOC-13 ^2^	0.018	0.4573	1.02	0.97	1.07
FIM ^3^ motor items	−0.017	0.5895	0.98	0.92	1.04
FIM ^3^ cognitive items	0.157	0.0693	1.17	0.99	1.40
Environmental factors *	1.484	0.0083	4.41	1.52	14.11

^1^ OGQ-J, Japanese version of the Occupational Gaps Questionnaire; ^2^ SOC-13, 13-item Sense of Coherence; ^3^ FIM, Functional Independence Measure; * *p* < 0.05; Model χ^2^, *p* < 0.001; Hosmer–Lemeshow, *p* = 0.232; The discriminant accuracy rate = 80.16%. Adjustment variable = age, sex, economic status.

## Data Availability

Not applicable.
